# Gender specialisation and stigma height dimorphism in Mediterranean *Lithodora fruticosa* (Boraginaceae)

**DOI:** 10.1111/plb.12634

**Published:** 2017-10-16

**Authors:** J. R. Pannell

**Affiliations:** ^1^ Department of Ecology and Evolution University of Lausanne Lausanne Switzerland

**Keywords:** Dioecy, distyly, functional gender, pollen limitation, sex allocation

## Abstract

- Dimorphism in style height has evolved repeatedly in flowering plants, with some individuals having short and others long styles; in the case of distylous species, stigma position varies reciprocally with that of the anthers. Distyly can be associated with divergence in the functional gender between long‐ and short‐styled individuals, but gender divergence has rarely been investigated in species with a simple stigma height polymorphism in the absence of reciprocal dimorphism in anther position.
- To evaluate the relation between stigma height polymorphism and gender, I measured the dimensions of floral morphology and seed production for the two morphs of a large population of the Iberian species *Lithodora fruticosa* (Boraginaceae).
- Results confirm the existence of a stigma height polymorphism in *L. fruticosa*, with long‐ and short‐styled individuals at a 1:1 ratio in the studied population. Long‐styled individuals produced substantially more seeds than did short‐styled individuals, pointing to strong divergence in functional gender between the two morphs.
- The results of this study are puzzling in light of recent work that suggests that *L. fruticosa* has a multi‐allelic self‐incompatibility system. I discuss the significance of gender divergence in *L. fruticosa* and evaluate hypotheses that might explain it.

## Introduction

The vast majority of angiosperms are hermaphroditic, with individuals transmitting their genes to progeny *via* both male and female functions (Renner & Ricklefs 1995; Renner 2014). Because each progeny has exactly one mother and one father, hermaphrodites must on average transmit genes to the next generation equally *via* their two genders, *i.e*., on a linear gender scale from zero (completely male) to one (completely female), individuals of any population will have a mean gender of 0.5 (Lloyd 1974, 1980). This characterisation of plant gender as a continuous, quantitative trait stands as one of the major advances in our quest to understand the diversity of plant sexual strategies (Lloyd 1980). Not only did it allow a move away from the fixed categories of ‘male’, ‘female’, which are often blurred in plants because of their often ‘leaky’ or inconstant gender expression (Lloyd & Bawa 1984; Ehlers & Bataillon 2007), but also to accommodate variation in the functional gender of individuals even in populations in which all individuals have both genders but vary in the extent to which they emphasise one gender over the other (Lloyd 1980).

The extent to which individuals in a hermaphroditic population deviate from the expected gender of 0.5 can be appreciated in terms of their ‘phenotypic’ quantitative gender, or in terms of their ‘functional’ gender (Lloyd 1980). A plant's phenotypic gender describes its allocation of resources to one sexual function relative to the other, while its functional gender reflects not so much its investment strategy, but its actual success as a male *versus* female parent. Thus, plants that transmit more of their genes to progeny *via* their pollen than their ovules will have a male‐biased functional gender (*i.e.,* a gender < 0.5). This bias may reflect a male‐biased phenotypic gender or sex allocation, but it may also simply be the outcome of who mates with whom, *i.e*., because the individual happens to be a better than average sire. Similarly, a hermaphrodite may be functionally more female than male not through an adapted sex allocation strategy but simply because its seed production is less strongly pollen‐limited. Hermaphrodite individuals that vary in their floral morphology, such as those in distylous populations, may also vary in this way in their phenotypic and/or functional gender.

Distyly is a hermaphroditic genetic polymorphism in which flowers are characterised by ‘reciprocal herkogamy’ in the placement of stigmas and anthers, with (usually) half the individuals having long styles and low‐level anthers, and (usually) the other half having short styles and high‐level anthers (Darwin 1877; Ganders 1979; Barrett 1992a). In species with a stigma height polymorphism, the two classes of hermaphrodites differ in the position of their stigmas in the floral tube, but the placement of their anthers tends to be similar in both morphs (reviewed in Barrett *et al*. 2000). In clades with both distylous species and those with a stigma height polymorphism, it is thought that the latter may give rise to the former, *i.e*., that distyly evolves by the spread of mutations that alter stigma position prior to those (at linked loci) that alter anther position and the states of ancillary traits (Barrett *et al*. 2000). Individuals in distylous species may also vary in their functional gender, with (usually) long‐styled plants showing a tendency towards an enhanced female function, and (usually) short‐styled plants being consequently more male in their gender (Hamilton 1990; Richards & Koptur 1993; Faivre & McDade 2001; Li *et al*. 2010). Indeed, distyly is one of the hypothesised evolutionary routes to dioecy, which might evolve *via* the gradual feminisation and masculinisation of long‐styled and short‐styled individuals, respectively (reviewed in Ganders 1979; Beach & Bawa 1980; Li *et al*. 2010). *Mussaenda pubescens*, which displays a stigma height dimorphism, is cryptically dioecious, with long‐ and short‐styled morphs being functionally fully female and fully male, respectively (Li *et al*. 2010). However, little is known about partial gender divergence between the morphs of species with a simple stylar dimorphism, which could also be an early stage in the evolution of fully separate sexes.

In this article, I present evidence for a divergence in gender between morphs of large population of the Mediterranean species *Lithodora fruticosa* (Boraginaceae), a small woody shrub that displays a stylar height dimorphism (Ferrero *et al*. 2009, 2011a,b,c, 2017). In contrast to *M. pubescens* just cited (Li *et al*. 2010), preliminary observations suggest that the gender dimorphism in *L. fruticosa* is only partial: although long‐styled individuals produce more seeds than short‐styled individuals, both morphs contribute genes to the next generation through both their sexual functions. My study has two principal aims: (i) to describe the variation in floral morphology presented by a large population of *L. fruticosa* with a view to formulating hypotheses concerning likely patterns of mating; and (ii) to estimate divergence in functional gender on the basis of observations of the distribution of fruit and seed set between and within the two stylar morphs.

To describe the floral variation in *L. fruticosa* and to characterise its potential bimodality in gender, I sampled flowers from a single population of the species in eastern Spain and measured their floral tubes, the position of their anthers and the height of their styles and thus the position of their stigmas. I estimated the female component of gender of individuals of the different style morphs observed by counting the numbers of seeds produced per fruit. *L. fruticosa* produces a single ovule in each of the locules of two fused bi‐locular carpels, with fruits thus producing one, two, three or four seeds that are easily visible in mature fruits. Although Ferrero *et al*. (2011c) found no differences in the pollen production of the two morphs, a difference in the seed set of long‐ *versus* short‐styled plants can reflect a bias in their average functional gender, as explained in the Discussion.

## Material and Methods

### Study population and floral measurements

At the time of full flowering, in April 1999, I collected and fixed in 70% alcohol a single flower from each of 226 individuals in a population of several hundred individuals of *L. fruticosa* that was located on a limestone ridge northwest of Caravaca de la Cruz in southeast Spain (38°08′39.4′′ N; 1°54′38′′ W). For each flower sampled, I later measured the corolla diameter, the distance between the base of the flower and the top of the floral tube, the position of each anther, and the top of the style. Note that I measured the length of the floral tube as a reference point for the other measurements, whereas Ferrero *et al*. (2017) measured the full corolla length rather than diameter and floral tube length.

The position of the stigma and the five anthers per flower were analysed as the distance from the top of the floral tube, in both absolute and relative terms. Insects alight on the top of the flower and insert their proboscis into the floral tube; contact with the anthers and or stigma will thus be determined by the position of the sexual parts in relation to the length of the floral tube. For the absolute measure, I subtracted the length of the floral tube from the height of the stigma and that of the centre of each of the five anthers (measured from the base of the floral tube). For the relative measure, I divided stigma and anther heights by the length of the floral tube. Values >0 and >1 represent positions above the floral tube for the absolute and relative measures, respectively, while those <0 and <1 represent positions within the tube. For the purposes of analysis, long‐ and short‐styled plants were defined as those with their stigma height above and below the floral tube opening, respectively. The anthers were held uniformly at the floral tube opening.

### Measures of female reproductive success

In May 1999, when all plants were in fruit, I returned to the study population and counted the number of seeds in fruits for an average of 147 (range: 76–220) flowers on each of eight clearly long‐ and eight clearly short‐styled plants that were chosen haphazardly in the population from each morph class. Note that although the floral tubes drop off the plant after flowering, the styles of *L. fruticosa* remain intact so that fruits located on the plant can be associated with a morph phenotype (short or long).

### Data analysis

Dimensions of floral morphology (corolla width and style length) were analysed by linear regression against corolla tube length, with morph type (long‐styled *versus* short‐styled individuals, defined as having style length < or > corolla tube length, respectively) as a fixed categorical factor and floral dimensions treated as continuous factors. The distribution of stigma and anther positions was analysed graphically by plotting each trait in a sequence ranked on style length relative to the corolla tube opening. Deviation from 1:1 of the morph ratio (again calculated by tallying individuals in terms of their style length < or > corolla tube length for short‐ *versus* long‐styled individuals, respectively) was tested using a *χ* ^2^ test. Fruit set (the number of flowers developing into a fruit), and the mean number of seeds per fruit produced by individuals of the two morphs, were analysed using *t*‐tests.

## Results

### Floral morphology

**Figure 1:**
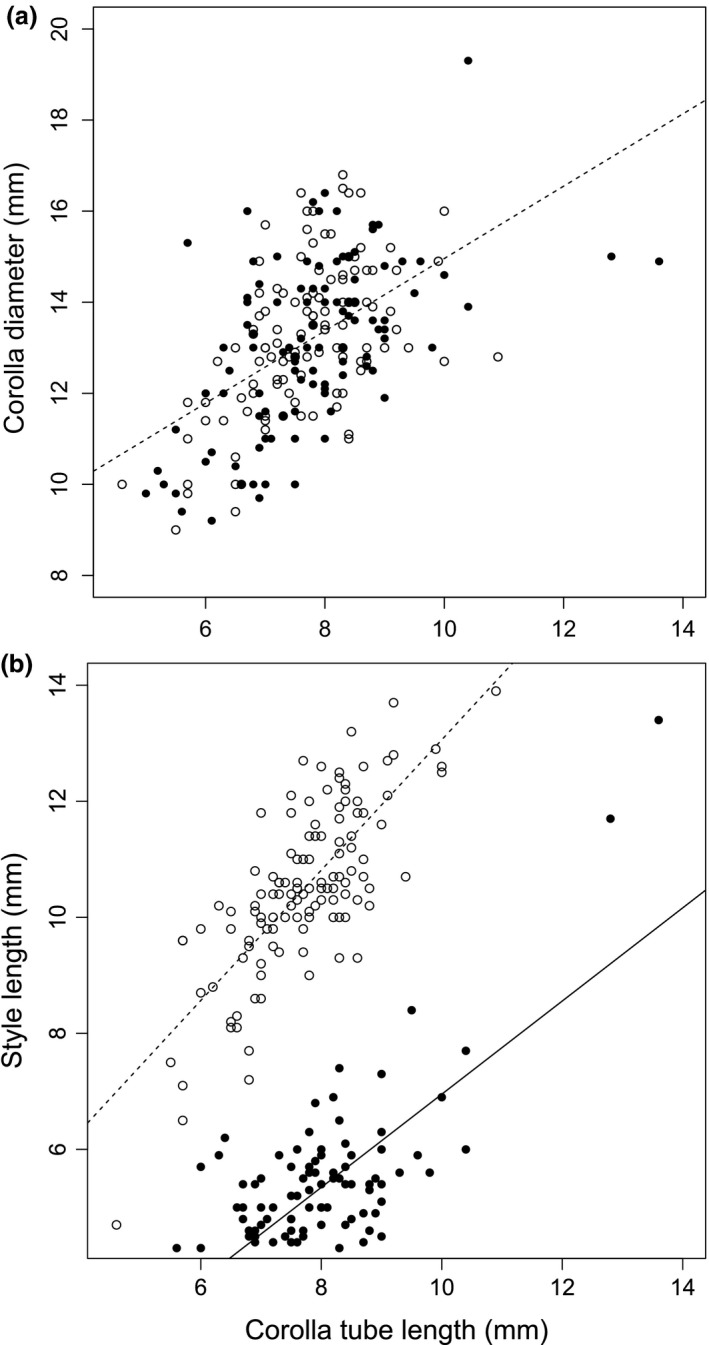
Relationship of a: corolla diameter and b: style length with corolla tube length in flowers of *Lithodora fruticosa*. Filled and unfilled circles correspond to flowers from short‐ and long‐styled individuals, respectively. The lines are regressions through the respective cloud of points (see text for details).

There was an allometric relationship among the principal dimensions of flower size. Corolla diameter was a positive function of corolla tube length, with floral diameter (mm) = 7.02 + 0.65 × tube length (*F*_1,222_ = 91.9, *P *<* *0.0001), a relationship that accounted for 29% of the variance in corolla width and which did not differ between the two morphs (morph × corolla length interaction term: *F*_1,222_ = 0.32, *P *=* *0.57; Fig. 1a). In contrast, style length was strongly associated with both corolla tube length and floral morph (morph × corolla length interaction: *F*_1,222_ = 7.92, *P *=* *0.0053; Fig. 1b). Unsurprisingly, long‐styled morphs had longer styles than short‐styled morphs, with morph category accounting for 76.3% of the variance in style length in the model. However, within each morph, style length also depended positively on floral tube length; for the short morph: style length (mm) = −1.1 + 0.8 × tube length; for the long morph: style length (mm) = 1.8 + 1.1 × tube length. These relationships accounted for 55% and 58% of the variance recorded for the short and long morphs, respectively (Fig. 1b).

**Figure 2:**
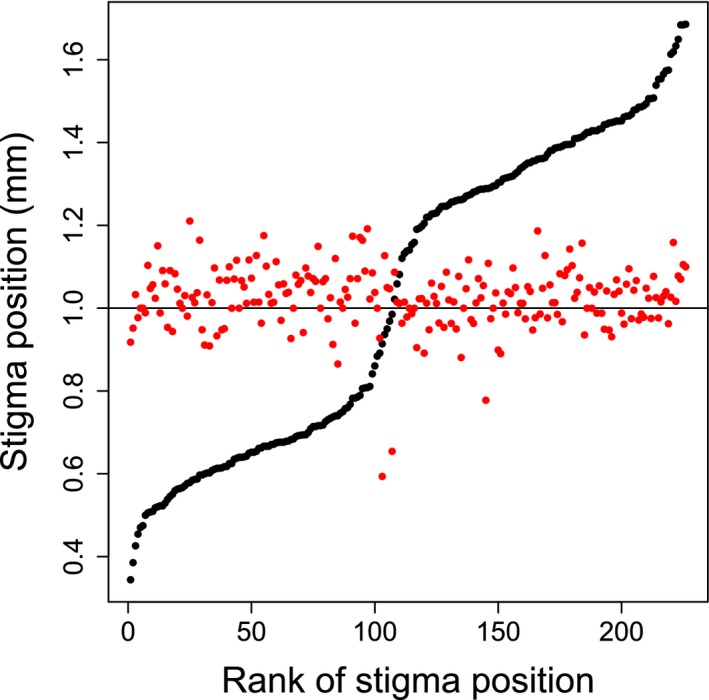
Distribution of style lengths (black points) and mid‐anther positions (red points; see text for details) in *Lithodora fruticosa*. Style lengths are plotted against their rank across the sampled population, with lengths given relative to the top of the corolla tube (indicated by the line at height = 1.0). Anther positions are plotted accordingly, *i.e.,* also relative to the top of the respective corolla tube. The morph designations for long‐ and short‐styled plants are indicated.

The distribution of the stigma and anther positions within flowers of *L. fruticosa* is shown in Fig. 2. While stigma positions (or style lengths) show a continuous distribution, the distribution is also clearly bimodal, with few individuals having stigmas in its central range (around the opening of the corolla tube). In contrast to the style length, the location of anthers within or above the floral tube opening depended very little on morph identity. Although anther positions differed significantly between morphs (morph × corolla tube length interaction: *F*_1,222_ = 8.21, *P *<* *0.01), morph accounted for only 5.1% of the variance in relative location (or 3.9% in a model that excluded corolla tube length as a factor), with 68% of the variance accounted for by the residual (*i.e.,* variance among individuals within a morph, and for flowers of the same corolla tube length; Fig. 2).

### Morph ratio

Taking the corolla tube opening as the point of reference for the centre of the distribution (corolla position = 1.0 in Fig. 2), with short‐ and long‐styled individuals having their styles below and above this point, respectively, there were 107 short‐styled plants and 119 long‐styled plants in the population, a ratio that is not significantly different from 1:1 (χ^2^_1_ = 0.64, *P* = 0.42).

### Fruit and seed production

**Figure 3:**
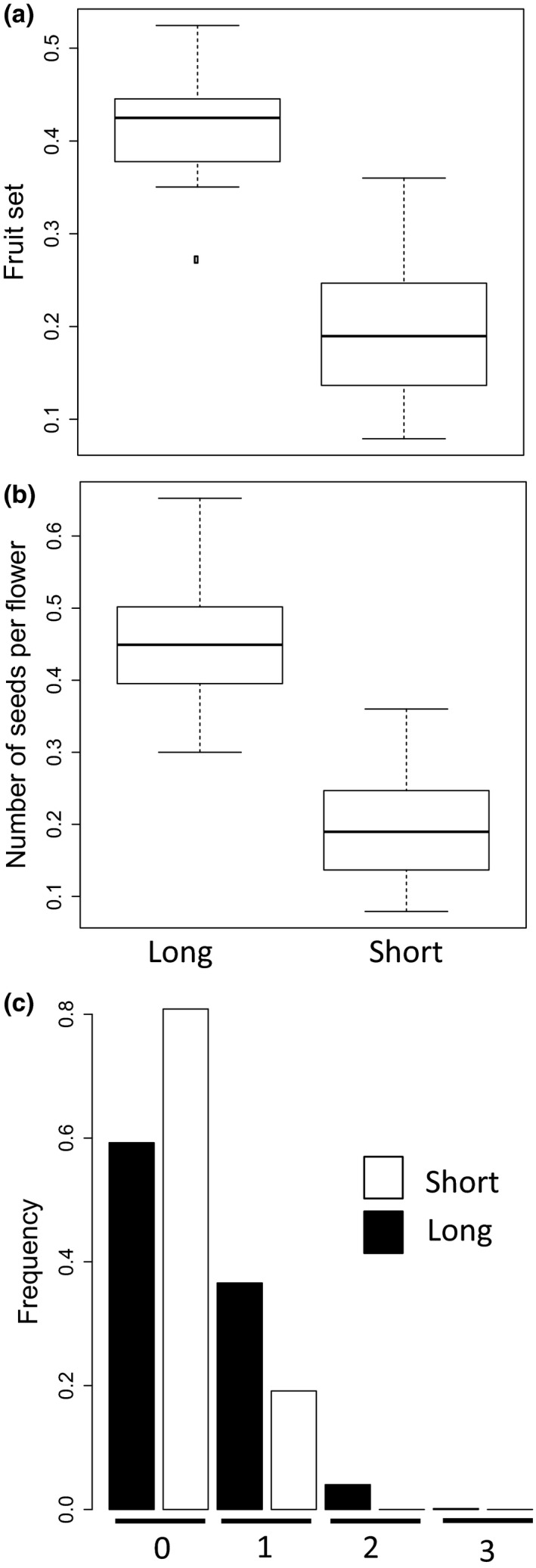
Female reproductive success of long‐styled (Long) and short‐styled (Short) plants of *Lithodora fruticosa*: a: fruit set (*i.e.,* fruits produced per flower); b: number of seeds per flower; and c: the frequency distribution of zero‐, one‐, two‐, and three‐seeded fruits. The box plots in (a) and (b) show the median (horizontal line), the 25% and 75% percentiles (box bottom and top), and the max and minimum values (whiskers), excluding outcliers (points). In (c), the proportions of flowers yielding different numbers of seeds is given separately for short‐styled (white) and long‐styled plants (black).

Long‐styled plants produced significantly more fruits per flower than short‐styled plants (*F*_1,14_ = 27.1, *P *<* *0.001), with morph accounting for 66% of the variance in fruits per flower (Fig. 3a). Similarly, long‐styled plants produced more seeds per flower (*F*_1,14_ = 22.2, *P *<* *0.001), with morph accounting for 67% of the variance in seed production (Fig. 3b). Specifically, long‐styled and short‐styled individuals produced 0.456 ± 0.039 and 0.198 ± 0.034 seeds per flower, respectively (Fig. 3b). Assuming that the two morphs produced the same number of flowers (see Discussion), long‐styled plants produced an average of 2.3 times more seeds than short‐styled plants.

Ovaries of *L. fruticosa* contain four ovules, so that the maximum number of seeds per fruit is also four. The distribution of the number of seeds per flower (and fruit) observed in the sample is shown in Fig. 3c. Of the 2,345 flowers assayed in total, none developed into a fruit with four seeds, and 68.5% of them had no seeds at all. Comparing the two morphs, 81% and 59% of flowers produced by short‐styled and long‐styled individuals, respectively, were seedless (see Fig. 3c).

## Discussion

### Stigma height dimorphism and flower‐size variation

The population of *L. fruticosa* studied here shows dimorphism in style length, with individuals having either long or short styles. Stigma height is not associated with reciprocal dimorphism in the position of anthers, which are placed at the same position at the opening of the floral tube in both morphs. These observations partly confirm conclusions reached in previous work on the species (Ferrero *et al*. 2011a,c, 2017), which also point to stigma height dimorphism as ancestral to reciprocal herkogamy (distyly) found elsewhere in the genus (Ferrero *et al*. 2009). Although floral‐tube length measured here was similar between the two morphs, within each morph deeper flowers were both somewhat wider and had longer styles, *i.e.,* there was a component of allometric variation in floral dimensions that was not associated with morph identity.

### Morph ratios

The population I studied was isoplethic, with the long‐styled and short‐styled morphs at similar frequencies. Interestingly, Ferrero *et al*. (2017) surveyed 22 populations of *L. fruticosa* in eastern Spain, of which only eight were isoplethic and 13 showed an excess of the long‐styled morph. Isoplethy in the population studied here is consistent with the maintenance of polymorphism with strongly (or even exclusively) disassortative mating. In contrast, the biased morph ratios found by Ferrero *et al*. (2017) suggest that, in the populations they observed with an excess of long‐styled plants, within‐morph mating occurred among long‐styled more frequently than among short‐styled individuals.

In many distylous species, disassortative mating is enforced through linkage between genes responsible for morphological differences between morphs and a bi‐allelic self‐incompatibility (SI) system, such that only inter‐morph mating is permitted (Barrett 1992b). I did not use crosses to test for inter‐ *versus* intra‐morph compatibility in the population studied here, but Ferrero *et al*. (2011a) did perform such crosses for *L. fruticosa* and concluded that the style‐length polymorphism was not linked to a diallelic self‐incompatibility (SI) system. If the same conclusion were to apply to the population studied here, and assuming that the observed 1:1 sex ratio I observed is not coincidental, the maintenance of isoplethy should be attributed entirely to a combination of disassortative mating promoted only by the morphological stylar dimorphism itself and, as discussed below, to a difference in functional gender between the two morphs. Given that neither anther positions nor pollen production (Ferrero *et al*. 2011c) differ between the two morphs of *L. fruticosa*, the greater seed set of the long‐styled morph in the absence of strong disassortative mating would presumably imply more frequent mating between long‐styled that between short‐styled individuals. However, that would in turn imply an equilibrium morph ratio biased towards the long‐styled morph, which I did not observe.

### Seed set and fruit set

Seed set and fruit set were low for both morphs of *L. fruticosa*, but particularly for the short‐styled morph. Indeed, approximately 80% and 60% of flowers produced by short‐styled and long‐styled individuals, respectively, yielded no fruits or seeds, and none of the >2,300 flowers sampled yielded four seeds, *i.e.,* the number of ovules expected in each ovary. Pollen supplementation experiments would be needed to ascertain the extent to which these low levels of fruit and seed set can be attributed to pollen limitation (Ashman *et al*. 2004). Such experiments were performed by Ferrero *et al*. (2011a), who found that seed set was low, even with inter‐morph pollen supplementation. Moreover, Ferrero *et al*. (2011b) reported high visitation rates to *L. fruticosa*, suggesting that pollen limitation is likely not sufficient to explain the low seed set in the species.

To the extent that the low seed set I observed here is indeed partially caused by pollen limitation, the strong difference in seed production between the two morphs might suggest that the short‐styled morph was more pollen‐limited than the long‐styled morph. This is plausible from the perspective of pollen receipt, as pollinators are more likely to touch the exserted stigmas of the long‐styled morph than those of the short‐styled morph that are held deep in the floral tube (Beach & Bawa 1980; Simón‐Porcar *et al*. 2015). However, we should expect to see a 1:1 morph ratio, as observed here, only if the short‐styled plants could compensate for their low seed set through correspondingly greater siring success. Yet short‐ and long‐styled plants produce the same number of pollen grains (Ferrero *et al*. 2011c), and liberate them from the same position at the top of the floral tube.

How else might we explain the 1:1 morph ratio observed, if it is not simply an outcome of chance? If greater siring success for the short‐styled morph were guaranteed by a diallelic self‐incompatibility (SI) system, as is common in distylous species, the 1:1 morph ratio would immediately be explained (*e.g.,* Larson & Barrett 1998). In this sense, the conclusion that *L. fruticosa* lacks a biallelic SI system with intra‐morph incompatibility (Ferrero *et al*. 2011a) is difficult to reconcile with my observations. In the absence of diallelic SI, the combination of a 1:1 morph ratio and strongly morph‐biased seed set could be explained either if pollen of the long‐styled morph were partly incapacitated by reduced fertility or if the long style interfered with pollen dispersal. To my knowledge, there is no evidence suggesting that pollen from the short‐styled plants is less capable of fertilising ovules than that from long‐styled plants. However, the potential for the position of the style or stigma to compromise pollen removal (Lloyd & Webb 1986) has been shown in a number of studies (*e.g.,* Fetscher 2001; Li *et al*. 2001; Sun *et al*. 2011; Guo *et al*. 2014; reviewed in Barrett 2002). Whether such an explanation might apply to *L. fruticosa* would need to be tested experimentally (*e.g.,* Sun *et al*. 2011). Of course it is possible, if *a priori* unlikely, that the population I studied differs in its SI system from those studied by Ferrero *et al*. (2017), *i.e.,* that is has biallelic SI, after all.

### Divergence in gender between morphs

The differences in seed set I observed between the long‐ and the short‐styled morph of *L. fruticosa* point to a clear divergence in functional gender between them, too. Although I did not estimate the amount of pollen produced by the two morphs, it is possible to calculate the mean functional gender of each morph directly from the seed data if we assume either intra‐morph incompatibility (so that all seeds produced by one morph are necessarily sired by the other), or simply that the observed isoplethy represents an equilibrium condition (which would imply equivalently strong disassortative mating), following Lloyd (1980). Specifically, Lloyd (1980) computed the prospective functional gender of the *i*th individual in a population as

$$G_{i}=\frac{g_{i}}{g_{i}+{\text{Ep}}_{i}}$$

where g_i_ and p_i_ are, respectively, the numbers of ovules (or countable female units, such as seeds or fruits) and pollen grains (or *e.g.,* anthers or male flowers) produced by the individual, and $E=\sum g_{j}/\sum p_{j}$ is an equivalence factor, that calibrates male and female allocations against one another, with the sums taken over all individuals of the population. G_i_ can be interpreted as a plant's ‘prospective’ femaleness, based on its investment in male *versus* female function relative to the investments made by everyone else.

Recall that the long‐styled morph produced 2.3‐fold more seeds per flower than the short‐styled morph. Assuming that each morph produces approximately the same number of flowers, we can set g_L_ = 2.3 and Ep_L_ = 1 for a representative long‐styled individual, L, in Lloyd's (1980) expression above. Accordingly, the functional femaleness of the long‐styled morph, on a scale from 0 to 1, is approximately 0.70. With a morph ratio of 1:1 and assuming strong disassortative mating (such that all seeds have one parent from each of the two morphs), the functional femaleness of the short‐styled morph should therefore be 0.3. The divergence in quantitative gender of the two morphs in the sampled population of *L. fruticosa* is thus substantial.

What mechanism might underlie the divergence in gender between the two morphs? One possibility is that the greater seed set of individuals of the long‐styled morphs is attributable simply to greater pollen receipt by their exserted stigmas. As noted earlier, this explanation would be consistent with a 1:1 morph ratio only if there were very strong disassortative mating, *e.g.,* brought about by intra‐morph incompatibility. Another possibility is that the exserted styles of the long morph might interfere with pollen removal in a way not affecting the short‐styled morph, as discussed above. Alternatively, the greater seed set of the long‐styled morph might be due to an evolved divergence in allocation strategy between the two morphs, *e.g.,* with the short‐styled producing fewer ovules or developing fewer of them into seeds than the long‐styled morphs. In such a case, we would equivalently expect the long‐styled morph to invest less in its male function than the short‐styled morph. Surprisingly, this does not appear to be so (Ferrero *et al*. 2011c).

### Concluding remarks

The results of this study indicate that *L. fruticosa*, a species with a stylar dimorphism, shows divergence in functional gender between long‐ and short‐styled morphs. Differences in functional gender have been described in a number of distylous species, usually with the long‐styled morph tending towards greater femaleness than the short‐styled morph (Hamilton 1990; Richards & Koptur 1993; Faivre & McDade 2001; Li *et al*. 2010). Moreover, the gradual divergence in gender between morphs is thought to have led to the ultimate evolution of dioecy from distyly in a number of species reviewed in (reviewed in Ganders 1979; Li *et al*. 2010). Recently, Li *et al*. (2010) showed that the species *Mussaenda pubescens*, which has individuals that differ in style length, is in fact functionally dioecious, with female and male functions fully impaired in short‐ and long‐styled morphs, respectively. Whether the lower seed set in the short‐styled morph of *L. fruticosa* is partly due to physiologically or developmentally impaired function remains an open question worth addressing; the low seed set found even upon legitimate pollination by Ferrero *et al*. (2011a) suggests that this might indeed be the case.
